# Evaluation of Attitudes and Perceptions in Students about the Use of Artificial Intelligence in Dentistry

**DOI:** 10.3390/dj11050125

**Published:** 2023-05-05

**Authors:** Milan Karan-Romero, Rodrigo Ernesto Salazar-Gamarra, Ximena Alejandra Leon-Rios

**Affiliations:** 1 School of Dentistry, Faculty of Health Sciences, Universidad Peruana de Ciencias Aplicadas, Av. Alameda San Marcos, 11, Chorrillos 15067, Lima, Peru; 2 UNIP Postgraduation Dental School, Paulista University (UNIP), São Paulo 05347, SP, Brazil; 3 CTS 367 Research Group, Andalusian Research Plan, Junta de Andalucía (Spain), University of Granada, 18071 Granada, Spain

**Keywords:** artificial intelligence, machine learning, deep learning, dentistry, attitudes, perceptions

## Abstract

Background: The implementation of artificial intelligence brings with it a great change in health care, however, there is a discrepancy about the perceptions and attitudes that dental students present towards these new technologies. Methods: The study design was observational, descriptive, and cross-sectional. A total of 200 dental students who met the inclusion criteria were surveyed online. For the qualitative variables, descriptive statistical measures were obtained, such as absolute and relative frequencies. For the comparison of the main variables with the type of educational institution, sex and level of education, the chi-square test or Fisher′s exact test was used according to the established assumptions with a level of statistical significance of *p* < 0.05 and a confidence level of 95%. Results: The results indicated that 86% of the students surveyed agreed that artificial intelligence will lead to great advances in dentistry. However, 45% of the participants disagreed that artificial intelligence would replace dentists in the future. In addition, the respondents agreed that the use of artificial intelligence should be part of undergraduate and postgraduate studies with 67% and 72% agreement rates respectively. Conclusion: The attitudes and perceptions of the students indicate that 86% agreed that artificial intelligence will lead to great advances in dentistry. This suggests a bright future for the relationship between dentists and artificial intelligence.

## 1. Introduction

Intelligence is defined as the mental capacity to obtain and retain a wide variety of knowledge and problem-solving skills. Likewise, it includes the use of critical reasoning and constant learning in relation to lived experiences [1]. Artificial intelligence (AI) is an imitation, replication or simulation of human intelligence created by the field of science and engineering in the form of a behavior capable of thinking, learning, solving situations and making decisions, which is expressed through technological artifacts [2,3,4]. The effective functioning of AI is given by automatic learning, also known as Machine Learning, which provides the ability to learn autonomously, making it possible for software to classify and predict a result from a database [5]. In addition, this aforementioned system processing has been enhanced with the discovery of deep learning, a subset of machine learning composed of multiple algorithms, which allows AI to be self-taught by learning from its own experiences to perform tasks such as voice and/or image recognition [5,6].

The implementation of artificial intelligence brings forth a great change in health care, since it is driven using databases together with a rapid analysis of the collected information and considering the patient at hand, facilitating work and increasing the healthcare professional′s productivity [7,8]. On the other hand, the link between artificial intelligence and dentistry is based on the intention of professional dentists to provide better treatment to patients through the use of this new technology, reducing treatment time, cost and the possibility of errors in decision making [9]. AI helps dentists from the most basic tasks, such as the registration of a patient′s medical history, to more complex activities, such as the analysis of the information obtained in order to reach an accurate diagnosis, possible treatment options and the prognosis of each one. Likewise, it is used in auxiliary tests such as x-rays, identifying findings and interpreting them with a higher level of accuracy to provide accurate diagnoses in a short time [10]. Using clinical data and diagnostic images, AI has been used to diagnose a number of oral diseases, including dental caries, maxillary sinus diseases, periodontal diseases, diseases of the salivary glands, TMJ problems, and oral cancer [11].

Although still under development, AI models for implant type detection, success prediction, and design optimization have shown considerable promise [11,12]. To date, artificial intelligence is not capable of replacing the dental surgeon, but it is vitally important to recognize technological advances to improve dental practice in the future [12].

However, the use of artificial intelligence may be influenced by the perceptions and attitudes that dentists have with regards to this new technology [13]. Perception is the cognitive process of consciousness that consists of recognition, interpretation and meaning for the elaboration of judgments. Conversely, an attitude is the mental disposition of a person to develop certain behaviors [14].

There are several previous studies that have evaluated the variables of perception and attitude on the use of artificial intelligence in the health sciences. Among them, a study carried out in Turkey with 1103 dental students in which they agreed that AI will have an impact on dental practices in the future [15]. Another study carried out in the United Kingdom evaluated the variables of perception and attitudes in medical students in relation to the use of artificial intelligence in auxiliary exams such as x-rays. The result of the research indicates that 88% of students agree that AI will have an important and positive role in health care, while 48.3% of students expressed concern about the possibility of being replaced by AI, considering the success artificial intelligence may have [16]. As far as we are aware, to date, there are no studies that tackle the aforementioned issue in Latin America. We believe it is of vital importance to carry out an evaluation of the perception and attitude in students regarding the use of AI in dentistry, since these young future professionals must be prepared and informed to face this technological change. The results provided by the research could be used in the future to guide new studies involving AI and health sciences. For this reason, the objective of this study was to evaluate the students′ attitudes and perceptions of the use of artificial intelligence in dentistry by sex, year of study and education institution.

## 2. Materials and Methods

### 2.1. Study Design

The study was descriptive, observational, and cross-sectional.

### 2.2. Sample Size

The study population was made up of dental university students enrolled in different universities in the Metropolitan region of Lima, Peru, during the period 2021–2022. The sample size was obtained through a proportion estimation formula from a previous study in dental students, of which 12% indicated not being aware of the use of AI in dentistry. The required sample size was 163 members. However, a 60% email response rate was added to the sample; therefore, it was sent to 260 participants. The type of sampling was convenience non-probabilistic. The unit of analysis was each university student who met the inclusion and exclusion requirements of the research.

### 2.3. Inclusion and Exclusion Criteria

Students from the first to the fifth year of a private or public university who agreed to be part of the research project and accepted the informed consent were included. Only participants over 18 years of age, male or female, who were fluent in Spanish were considered. Participants who inadequately filled out the survey and dental students from other countries who were undergoing an international exchange in Peru at the time were excluded from the study.

### 2.4. Ethical Considerations

This research project was reviewed and approved by the Ethics and Research subcommittee of the Faculty of Health Sciences of the University of Peruvian university of applied sciences with approval code: CEI/579-07-2. Likewise, in this research the ethical principles were considered with the intention of respecting the willingness and confidentiality of the participants.

### 2.5. Assessment of Attitudes and Perceptions about the Use of AI in Dentistry

To record the data, an instrument validated through a committee of experts was used. After the judgment of these seven experts, Aiken′s V statistic was obtained with a value of 0.90, which indicates good content validity. A pilot test was carried out with distribution of the questionnaire approved by the expert judgment to 20 participants. These participants had 15 min to complete the questionnaire and once it was finished, the creation of the database began. Cronbach′s Alpha coefficient of 0.90 was obtained, which determined a high reliability of the instrument. In addition, the survey was taken again by the same group with a time difference of two weeks. The results were used to calculate the degree of concordance of the questions using the KAPPA value, which was 0.99.

The questionnaire has 22 questions and is structured into 3 sections: the first part of the questionnaire is made up of four questions about sociodemographic characteristics such as age, sex, year of study and university where they study. In the second part, the participants are asked three questions about sources of information on the use of artificial intelligence in daily life, basic knowledge about the principles of AI functioning and knowledge about the use of AI in dentistry. Regarding the third section, the respondents are presented with 15 statements and asked to define their level of agreement, by choosing one of the following values: disagree, do not know and agree.

### 2.6. Analysis of Data

For the univariate analysis, descriptive statistical measures were obtained as absolute and relative frequencies of the main qualitative variables: attitude regarding the use of AI in dentistry and perceptions on the use of AI in dentistry, as well as the aforementioned covariates: sex, marital status, level of education, institution of higher education, socioeconomic status, place of residence, source of information about the use of AI in daily life, basic knowledge about the working principle of AI, awareness of the use of AI in dentistry. Considering the quantitative variable age, the mean was used as a measure of central tendency and the standard deviation as a measure of dispersion.

To compare students′ attitudes and perceptions regarding the use of artificial intelligence in dentistry according to sex, level of education and higher educational institution, the chi-square test or Fisher′s exact test were used. In addition, the confidence level was 95% and the p value was set at less than 0.05 to determine the level of statistical significance. The program used to analyze the results was STATA® version 16.0 software (StataCorp, College Station, TX, USA).

## 3. Results

The purpose of this study was to evaluate the attitudes and perceptions of university students in Metropolitan Lima, Peru, regarding the use of artificial intelligence in dentistry. A total of 200 students who met the inclusion criteria were evaluated.

Table 1 presents the data on the general and sociodemographic characteristics of the participants. Of the study participants, 102 were men (51%) and 98 women (49%). The mean age of the participants was 22 years (2.87). Regarding the type of higher institution, the majority went to private universities, 155 (77.50%). In relation to the level of education, the most prominent category was the 5th year of studies with 74 respondents (37%), while the group with the fewest participants was the group of 1st year students with as few as 14 (7%). Finally, regarding the socioeconomic level, it was observed that 169 participants (84.50%) belonged to the high-level category.

Table 2 shows the evaluation of the attitudes and perceptions of university students in Lima about the use of AI in dentistry. It is observed that the vast majority of the participants, 172 (86%), agreed that artificial intelligence will lead to great advances in dentistry, while 90 students (45%) disagreed with the statement that artificial intelligence will replace dentists in the future and 42 (21%) were uncertain about this statement. Furthermore, 181 participants (90.5%) indicated that they were very excited about the use of this new technology in dentistry and medicine. Also, respondents agreed that the use of artificial intelligence should be part of undergraduate (67%) and postgraduate (72%) studies. On the other hand, the majority agreed regarding the use of artificial intelligence in the radiographic diagnosis of dental caries (85.50), soft tissue injuries (72.50%), maxillary pathologies (73%) and periodontal diseases (68%). Finally, there was a broad agreement of the participants (60.50%) that artificial intelligence could be used in forensic odontology.

Table 3 shows the comparison of the attitudes and perceptions of university students in Metropolitan Lima about the use of Artificial Intelligence according to the type of higher education institution, sex and level of education. We found that there was a significant difference between the two statements “AI will lead to great advances in dentistry and medicine” and “AI can be used as a quality control tool to evaluate the success of treatments”. Likewise, a significant difference was observed between women and men according to the statement “AI may replace dentists and doctors in the future”, where 62.22% of the women indicated that they did not agree, while 64.71% of the men agreed with that statement. It was concluded that women are more optimistic than men about the possibility of being replaced by this new technology in the future. Significant differences were also found between the following two statements “AI applications should be part of undergraduate dental education”, “AI can be used in forensic dentistry”. In both statements, a higher percentage of men disagreed with women with 68.09% and 65% respectively of men compared to 31.91% and 35% of women. On the other hand, in relation to the level of education, no statistically significant difference was found.

## 4. Discussion

The purpose of this study was to evaluate the attitudes and perceptions about the use of artificial intelligence in dentistry of university students in Metropolitan Lima, Peru. Two hundred people who met the inclusion criteria were evaluated. The results indicate that the majority of the participants agreed that artificial intelligence would have a real and positive impact on dental practice.

Artificial intelligence, as a possible technological innovation, has numerous uses in health care, particularly in dentistry [17,18]. In the present investigation, it was found that 86% of the participants agreed that AI will lead to great advances in dentistry and medicine in the future. This result is similar to the study by the author Seram Tampha in India during 2021, in which the majority of participants agreed that AI will be of great importance in dentistry in the near future [19]. This could be because artificial intelligence encompasses a broad spectrum of emerging technologies that continue to influence daily life. The evolution of AI makes big data analysis possible, providing reliable information and improving the decision-making process [20].

Our study found that 45% of the respondents did not believe that dentists would be replaced by artificial intelligence in the future. This result is similar to that obtained by Emir Yüzbaşıoğlu in a study carried out during 2020 in Turkey on dental students, in which 52.6% of the participants indicated that they disagreed with this same statement. Likewise, a study carried out in India in 2021, found that 37.78% of dental interns disagreed that they would be replaced by this new technology. [15]. Artificial intelligence is faced with a greater challenge to replace to dentists compared to other professions, due to the interaction that exists between the dental operator and the patient. A device with AI, unlike a person, does not have the ability to generate a high level of trust, calmness and/or empathy with the patient, which are essential characteristics in dental care [21,22].

It was observed that 76.5% of the participants in this research had an acceptance position regarding the use of artificial intelligence as a planning tool for dental diagnosis and treatment. A similar study conducted in Saudi Arabia by Sanjeev Khanagar in 2021 established that 67.6% of the participants indicated that they agreed with the aforementioned statement [23]. Artificial intelligence in the field of health has revolutionized accuracy in diagnosis and dental treatment planning, since these systems have the ability to simplify tasks and provide results in less time, which allows the dentist to save on resources and be more efficient in their dental practices [24,25].

Regarding the use of artificial intelligence in the radiographic diagnosis of dental caries, 85.50% of the students in our research project indicated that they agree that this technology can be used in that area. This result is like the study conducted in Turkey in which 84.2% of the participants agreed with the same statement [15]. This may be due to the high performance that neural network algorithms have shown in the radiographic diagnosis of dental caries, in molars and premolars, achieving a level of precision greater than 82%. In pediatric dentists, IA is used in clinical decision making, developing preventive strategies, and establishing an appropriate treatment plan [25,26,27].

Furthermore, statistically significant differences were found between attitudes and perceptions about the use of artificial intelligence in dentistry in relation to the type of higher institution and sex of the participant. One of the significant differences found was in the statement “AI can replace dentists and doctors in the future” according to sex, where the highest percentage of participants in agreement were men. This finding coincides with the results of a study carried out in India in which 28.57% of dental students and graduates agreed that AI could replace dentists or doctors [23]. This may lead us to think that dentists are more optimistic about the possibility of being replaced in the future. It is undeniable that machine learning has come to replace human labor in certain activities [24,27]. A Deloitte collaboration with the Oxford Martin Institute suggested that AI could automate 35% of jobs in the United Kingdom over the next 10–20 years [28]. However, it is known that in the field of healthcare this substitution is more difficult due to the ethical implications surrounding the use of AI in this sector. Therefore, AI systems are unlikely to supplant human physicians on a large scale, but rather join efforts in the care of patients [29].

With regards to the sex of the respondents, it was found that there was a significant difference in relation to the item: "Artificial intelligence should be part of undergraduate education" where we found that of the participants who rejected this statement, 31.91% were women. However, this result differs from that obtained by Khanagar S, who indicated in their study conducted in India that 75% of the participants who disagreed were women [23]. These results may be influenced by a cultural factor specific to each country that leads to greater inequality in education according to sex or gender [29]. Women seem to have a positive attitude towards learning and implementation of new techniques, however, globally, they continue to be underrepresented in this field [30].

On the other hand, it was observed that the presence of a significant difference compared to whether AI will lead to great advances in the future according to the type of higher institution, in which 80.23% of the university students who agreed were those who attended a private university. This may be due to a lack of knowledge about technology on behalf of the students at public universities due to the notable differences that exists in the resources allocated to universities, in which public universities receive funds from the state, while private ones use their own funds and resources leading to disparities in the access to better teachers and more modern technology [31].

One of the main limitations of our study is in relation to the method of data collection. When using a self-report questionnaire there is the possibility of social desirability bias on the part of the respondents, in which they complete the survey in a socially accepted manner rather than according to their own criteria. However, we believe this was offset by the reliability and validity obtained from the questionnaire [32].

Finally, this research project is important because, to date, there are no previous studies on the subject in Latin America, this being the first report of the attitudes and perceptions of Peruvian dental students about the use of AI in dental practice. Likewise, the resulting information could serve as a precedent to guide the development of future research following this same line. The results reflect that there is a general acceptance among the participants of artificial intelligence in dentistry, since most agree with the statements regarding its use in this sector.

In Peru the digital dentistry course is an elective course, so not all faculties offer such a course, however, if there is interest from students, the curriculum could be improved. This fact could support the implementation of informative workshops on AI in dentistry and evaluate the possibility of including AI training in the study programs and university curricula.

## Figures and Tables

**Table 1:** General Characteristics of university dental students surveyed in the region of Lima -Peru (*n* = 200).

Variable	*n*	%
Age of the Students in Years (SD) *	22. 22. 2.87 *	
**Sex**		
Masculine	102	51.00
Femenine	98	49.00
**Residence**		
Lima South	33	16.50
Lima North	31	15.50
Lima Center	94	47.00
Lima East	42	21.00
**Year of Study**		
1st year	14	7.00
2nd year	31	15.50
3rd year	32	16.00
4th year	49	24.50
5th year	74	37.00
**University Institution**		
Public	45	22.50
Private	155	77.50
**Socioeconomic Leve**		
Lower	3	1.50
Avarage	28	14.00
Higher	169	84.50

* Mean (desviation).

**Table 2:** Evaluation of the attitudes and perceptions of university students in Lima about the use of AI in education (*n* = 200).

Items	Disagree *n* (%)	I Don′t Know. *n* (%)	Agree *n* (%)
AI will lead to great advances in dentistry and medicine	10 (5.00)	18 (9.00)	172 (86.00)
AI may replace dentist and doctors in the future	90 (45.00)	42 (21.00)	68 (34.00)
AI can be used as a definitive diagnostic tool in disease diagnosis	27 (13.50)	36 (18.00)	137 (68.50)
AI can be used as a prognostic tool to predict the course of a disease and determine if there is a chance of recovery	14 (7.00)	33 (16.50)	153 (76.50)
AI can be used in the three-dimensional positioning and planning of implants	15 (7.50)	31 (15.50)	154 (77.00)
AI can be used as a treatment planning tool in the diagnosis and planning of dental treatment	15 (7.50)	32 (16.00)	153 (76.50)
AI can be used as a quality control tool to assess the success of treatments	13 (6.50)	28 (14.00)	159 (79.50)
AI applications should be part of undergraduate dental education	47 (23.50)	19 (9.50)	134 (67.00)
AI applications should be part of postgraduate dental education	32 (16.00)	24 (12.00)	144 (72.00)
The use of AI in dentistry and medicine is exciting	10 (5.00)	9 (4.50)	181 (90.50)
AI can be used for radiographic diagnosis of dental caries	12 (6.00)	17 (8.50)	171 (85.50)
AI can be used for the diagnosis of soft tissue injuries in the oral cavity	13 (6.50)	42 (21.00)	145 (72.50)
AI can be used for radiographic diagnosis of jaw pathologies	16 (8.00)	38 (19.00)	146 (73.00)
AI can be used for the radiographic diagnosis of periodontal diseases	19 (9.50)	45 (22.50)	136 (68.00)
AI can be used in forensic odontology	40 (20.00)	39 (19.50)	121 (60.50)

**Table 3:** Comparison of student′s attitudes and perceptions about the use of artificial intelligence in dentistry according to type of higher education institution, gender and level of education (*n* = 200).

Items	Private *n* (%)	Public *n* (%)	*p*	1st Year *n* (%)	2nd Year *n* (%)	3rd Year *n* (%)	4th Year *n* (%)	5th Year *n* (%)	*p*	Male	Female	*p*
AI will lead to great advances in dentistry and medicine.												
Agree	7 (70.00)	3 (30.00)	0.042	2 (20.00)	2 (20.00)	1 (10.00)	4 (40.00)	1 (10.00)	0.310 *	7 (70.00)	3 (30.00)	0.468 **
I don′t know.	10 (55.56)	8 (44–44)		1 (5.56)	4 (22.22)	1 (5.56)	5 (27.78)	7 (38.89)		9 (50.00)	9 (50.00)	
Disagree	138 (80.23)	34 (19.77)		11 (6.40)	25 (14.53)	30 (17.44)	40 (23.26)	66 (38.37)		86 (50.00)	86 (50.00)	
AI may replace dentist and doctors in the future.												
Agree	66 (73.33)	24 (26.67)	0.282 **	6 (6.67)	12 (13.33)	15 (16.67)	26 (28.89)	31 (34.44)	0.089 **	34 (37.78)	56 (62.22)	0.002 **
I don′t know.	36 (57.14)	6 (14.29)		2 (4.76)	3 (7.14)	4 (9.52)	14 (33.33)	19 (45.24)		24 (57.14)	18 (42.86)	
Disagree	53 (77.94)	15 (22.06)		6 (8.82)	16 (23.53)	13 (19.12)	9 (13.24)	24 (35.29)		44 (64.71)	24 (35.29)	
AI can be used as a definitive diagnostic tool in disease diagnosis.												
Agree	23 (85.19)	4 (14.81)	0.319 **	2 (7.41)	4 (14.81)	4 (14.81)	7 (25.93)	10 (37.04)	0.997 *	14 (51.85)	13 (48.15)	0.989 **
I don′t know.	25 (69.44)	11 (30.56)		3 (8.33)	7 (19.44)	5 (13.89)	9 (25.00)	12 (33.33)		18 (50.00)	18 (50.00)	
Disagree	107 (78.10)	30 (21.90)		9 (6.57)	20 (14.60)	23 (16.79)	33 (24.09)	52 (37.96)		70 (51.09)	67 (48.91)	
AI can be used as a prognostic tool to predict the course of a disease and determine if there is a chance of recovery.												
Agree	12 (85.71)	2 (14.29)	0.225 **	1 (7.14)	0 (0.00)	2 (14.29)	7 (50.00)	4 (28.57)	0.176 *	7 (50.00)	7 (50.00)	0.481 **
I don′t know.	22 (66.67)	11 (33.33)		4 (12.12)	8 (24.24)	6 (18.18)	6 (18.18)	9 (27.27)		20 (60.61)	13 (39.39)	
Disagree	121 (79.08)	32 (20.92)		9 (5.88)	23( 15.03)	24 (15.69)	36 (23.53)	61 (39.87)		75 (49.02)	78 (50.98)	
AI can be used in the three-dimensional positioning and planning of implants.												
Agree	13 (86.87)	2 (13.33)	0.053 **	1 (6.67)	1 (6.67)	2 (13.33)	6 (40.00)	5 (33.33)	0.271 *	6 (40.00)	9 (60.00)	0.206 **
I don′t know.	19 (61.19)	12 (38.71)		4 (12.90)	4 (12.90)	9 (29.03)	6 (19.35)	8 (25.81)		20 (64.52)	16 (35.48)	
Disagree	123 (79.87)	31 (20.13)		9 (5.84)	26 (16.88)	21 (13.64)	37 (24.03)	61 (39.61)		76 (49.35)	78 (50.65)	
AI can be used as a treatment planning tool in the diagnosis and planning of dental treatment												
Agree	12 (80.00)	3 (20.00)	0.086 **	2 (13.33)	1 (6.67)	1 (6.67)	8 (53.33)	3 (20.00)	0.131 *	7 (46.67)	8 (53.33)	0.928 **
I don′t know.	20 (62.50)	12 (37.50)		4 (12.50)	5 (15.63)	7 (21.88)	6 (18.75)	10 (31.25)		16 (50.00)	16 (50.00)	
Disagree	123 (80.39)	30 (19.61)		8 (5.23)	25 (16.34)	24 (15.69)	35 (22.88)	61 (39.87)		79 (51.63)	74 (48.37)	
AI can be used as a quality control tool to assess the success of treatments.												
Agree	12 (92.31)	1 (7.69)	0.040 **	2 (15.38)	1 (7.69)	3 (23.08)	5 (38.46)	2 (15.38)	0.243 *	5 (38.46)	8 (61.54)	0.631 **
I don′t know.	17 (60.71)	11 (39.29)		4 (14.29)	5 (17.86)	4 (14.29)	7 (25.00)	8 (28.57)		14 (50.00)	14 (50.00)	
Disagree	126 (79.25)	33 (20.75)		8 (5.03)	25 (15.72)	25 (15.72)	37 (13.27)	64 (40.25)		83 (52.20)	76 (47.80)	
AI applications should be part of undergraduate dental education.												
Agree	36 (76.70)	11 (23.40)	0.084 **	6 (12.77)	8 (17.02)	10 (21.28)	10 (21.28)	13 (27.66)	0.268 **	32 (68.09)	15 (31.91)	0.026 **
I don′t know.	11 (57.89)	8 (42.11)		3 (15.79)	2 (10.53)	3 (15.79)	5 (26.32)	6 (31.58)		8 (42.11)	11 (57.89)	
Disagree	108 (80.60)	26 (19.40)		5 (3.73)	21 (15.67)	19 (14.18)	34 (25.37)	55 (41.04)		62 (46.27)	72 (53.73)	
AI applications should be part of postgraduate dental education.												
Agree	24 (75.00)	8 (25.00)	0.334 **	5 (15.63)	5 (15.63)	9 (28.13)	6 (18.75)	7 (21.88)	0.095 **	19 (59.38)	13 (40.63)	0.545 **
I don′t know.	16 (66.67)	8 (33.33)		1 (4.17)	5 (20.83)	4 (16.67)	8 (33.33)	6 (25.00)		11 (45.83)	13 (54.17)	
Disagree	115 (79.86)	29 (20.14)		8 (5.56)	21 (14.58)	19 (13.19)	35 (24.31)	61 (42.36)		72 (50.00)	72 (50.00)	
The use of AI in dentistry and medicine is exciting.												
Agree	7 (70.00)	3 (30.00)	0.617 *	2 (20.00)	0 (0.00)	1 (10.00)	1 (10.00)	6 (60.00)	0.134 *	5 (50.00)	5 (50.00)	0.621 *
I don′t know.	8 (88.89)	1 (11.11)		0 (0.00)	3 (33.33)	0 (0.00)	4 (44.44)	2 (22.22)		3 (33.33)	6 (66.67)	
Disagree	140 (77.35)	41 (22.65)		12 (6.63)	28 (15.47)	31 (17.13)	44 (24.31)	66 (36.46)		84 (51.93)	87 (48.07)	
AI can be used for radiographic diagnosis of dental caries.												
Agree	7 (58.33)	5 (41.67)	0.090 *	1 (8.33)	2 (16.67)	1 (8.33)	2 (16.67)	6 (50.00)	0.227 *	6 (50.00)	6 (50.00)	0.939 **
I don′t know.	11 (64.71)	6 (35.29)		0 (0.00)	0 (0.00)	4 (23.53)	4 (23.53)	9 (52.94)		8 (47.06)	9 (52.94)	
Disagree	137 (80.12)	34 (19.88)		13 (7.60)	29 (16.96)	27 (15.79)	43 (25.15)	59 (34.00)		88 (51.46)	83 (48.54)	
AI can be used for the diagnosis of soft tissue injuries in the oral cavity.												
Agree	9 (69.23)	4 (30.77)	0.721 **	1 (7.69)	3 (23.08)	1 (7.69)	2 (15.38)	6 (50.00)	0.768 *	4 (30.77)	9 (69.23)	0.185 **
I don′t know.	32 (76.19)	10 (23.81)		3 (7.14)	6 (14.29)	7 (16.67)	9 (21.43)	17 (40.48)		25 (59.52)	17 (40.48)	
Disagree	114 (78.62)	31 (21.38)		10 (6.90)	22 (15.17)	24 (16.55)	38 (26.21)	51 (35.17)		73 (50.34)	72 (49.66)	
AI can be used for radiographic diagnosis of jaw pathologies.												
Agree	11 (68.75)	5 (31.25)	0.512 **	2 (12.50)	4 (25.00)	2 (12.50)	3 (18.75)	5 (31.25)	0.964 *	6 (37.50)	10 (62.50)	0.507 **
I don′t know.	28 (73.68)	10 (26.32)		1 (2.63)	4 (10.53)	7 (18.42)	12 (31.58)	14 (36.84)		19 (50.00)	19 (50.00)	
Disagree	116 (79.45)	30 (20.55)		11 (7.53)	23 (15.75)	23 (15.75)	34 (23.29)	55 (37.67)		77 (52.74)	69 (47.26)	
AI can be used for the radiographic diagnosis of periodontal diseases.												
Agree	13 (68.42)	6 (31.58)	0.604 **	2 (10.53)	4 (21.05)	4 (21.05)	2 (10.53)	7 (36.84)	0.822 *	11 (57.89)	8 (42.11)	0.865 **
I don′t know.	35 (77.78)	10 (22.22)		2 (4.44)	7 (15.56)	8 (17.78)	10 (22.22)	18 (40.00)		21 (46.67)	24 (53.33)	
Disagree	107 (78.68)	29 (21.32)		10 (7.35)	20 (14.71)	20 (14.71)	37 (27.21)	49 (36.03)		70 (51.47)	68 (48.53)	
AI can be used in forensic odontology.												
Agree	31 (77.50)	9 (22.50)	0.864 **	4 (10.00)	10 (25.00)	11 (27.50)	5 (12.50)	10 (25.00)	0.108 **	26 (65.00)	14 (35.00)	0.036 **
I don′t know.	29 (74.36)	10 (25.64)		2 (5.13)	6 (15.38)	6 (15.38)	10 (25.64)	15 (38.46)		23 (58.97)	16 (41.03)	
Disagree	95 (78.51)	26 (21.49)		8 (6.61)	15 (12.40)	15 (12.40)	34 (28.10)	49 (40.50)		53 (43.80)	68 (56.20)	

*: Fisher′s exact test, **: Chi-square test, *p* < 0.05.

## Data Availability

The data that support the findings of this study are available on request from the corresponding author. The data are not publicly available due to privacy or ethical restrictions.
